# Non-Adherence to Peripheral Venous Catheter Care Protocols Significantly Decreases Patient Safety and Impacts Costs: A Retrospective Observational Study

**DOI:** 10.3390/healthcare12161558

**Published:** 2024-08-06

**Authors:** María Bibiano Guillén, Jose Tolsdorf Rodríguez, Javier Nuñez-Alfonsel, José Miguel Cárdenas-Rebollo, Ángel Ayuso-Sacido

**Affiliations:** 1 Quality Department Nurse, HM Hospitales, 28015 Madrid, Spain; 2 Quality and ESG Department, HM Hospitales, 28015 Madrid, Spain; jtolsdorf@hmhospitales.com; 3 Clinical Efficiency Validation Institute (IVEc), HM Research Foundation (FiHM), 28015 Madrid, Spain; jnalfonsel@fundacionhm.com; 4 Mathematics and Data Science Department, San Pablo CEU University, 28660 Madrid, Spain; 5 Nursing Department, San Pablo CEU University, 28660 Madrid, Spain; 6 Brain Tumor Laboratory, Foundation Vithas, Vithas Hospital Group, 28043 Madrid, Spain; 7 Faculty of Experimental Sciences and Faculty of Medicine, Francisco de Vitoria University, 28223 Madrid, Spain

**Keywords:** adherence, NANDA-I, nursing interventions, patient safety, peripheral venous catheter, protocol, quality, cost

## Abstract

In the healthcare field, the effective implementation of clinical protocols is crucial to ensuring patient safety and well-being. In this context, this study evaluates nurses’ adherence to the maintenance and replacement protocol of peripheral venous catheters (PVCs) in a university hospital in Spain, examining the impact of compliance with the protocol on the loss of PVCs and on patient safety in addition to analyzing the related costs. A retrospective observational study was conducted with 590 patients who were admitted in 2018 and 2019. The chi-square test or Fisher’s exact test, as appropriate, was used to see the association between the study variables; with the dependent variable being the loss of PVCs (including, as a dependent variable, the loss of PVCs before 48 h). The patients’ electronic and physical medical records were reviewed to analyze nursing interventions related to the management of PVCs. A total of 24% of patients experienced PVC loss within the first 24 h after insertion. Failure to comply with the protocol resulted in 80% more catheter loss and increased the cost of cannulation by 46.84%. Low compliance with PVC care protocols significantly increases the risk of catheter loss, suggesting the need for increased training and strict protocol implementation. The findings emphasize the critical role of nursing in ensuring patient safety through adherence to evidence-based protocols. Continuing education and diligent protocol implementation are essential to reducing healthcare costs and improving patient outcomes.

## 1. Introduction

In 2004, the World Health Organization (WHO) established the Global Patient Safety Alliance with the general aim of “Do No Harm” during health care, and, for this, it identified, as a common denominator, 10 lines of action for all participants of this Alliance [1]. The objective is to eliminate or reduce, to reasonable minimum levels, the preventable damage derived from patient care.

Despite healthcare having a significant impact on patient safety, the outcomes remain disheartening. According to the 2021 Prevalence Study of Nosocomial Infections (EPINE) in Spain, 1 in 10 hospitalized patients experience preventable harm, with at least 50% of these cases being avoidable [2].

In the context of nursing care, the insertion and maintenance of the peripheral venous catheter (PVC) and adherence to established protocols are crucial, constituting a routine part of nursing practice. It is crucial to consistently apply standard precautions in the management of PVCs, such as proper selection of the insertion site, maintenance of aseptic techniques during insertion, and constant monitoring of the insertion site to detect signs of infection or complications [1,3,4]. Most patients in emergency departments or hospital admissions receive a PVC. This is substantiated by the EPINE 2021 study, which indicates that 76.57% (of 49,836 patients) were fitted with a catheter [2].

Various studies and approaches exist regarding the protocols for insertion and maintenance of PVCs, yielding diverse outcomes. Estimates suggest that the incidence of phlebitis can range between 16% and 55% in the absence of preventive measures [1,3,4,5]. However, assessing patient safety costs during hospitalization, based on adherence to these protocols, offers a valuable tool for decision-making and healthcare system management. It is crucial to evaluate the actual impact of compliance with PVC management protocols on catheter loss and, consequently, on reducing potential harm and adverse effects to the patient, thereby enhancing patient safety. Also, we highlight the increase in costs. The loss of PVC implies added costs for its replacement, including materials and nursing time, increasing hospital spending [1,6]. To investigate this issue, the study aimed to assess the loss of adherence, by nurses, to the previously developed and implemented established protocol for PVC management and its subsequent impact on patient safety, in addition to analyzing the related costs.

## 2. Materials and Methods

### 2.1. Design and Configuration of the Study

A retrospective observational descriptive study of 603 patients admitted to the HM Torrelodones University Hospital, in Madrid (Spain) was performed during the years 2018 and 2019, of which 11 patients were excluded, leaving a final sample of 590 patients.

### 2.2. Subjects

The study’s patient selection criteria included those admitted to HM Torrelodones University Hospital under Internal Medicine or Traumatology services, aged 65 years or older, of either male or female gender, with a median hospital stay of 10 days or more. Exclusion criteria encompassed patients without a peripheral venous catheter and those with catheters inserted at other hospitals or healthcare centers.

**Figure 1:**
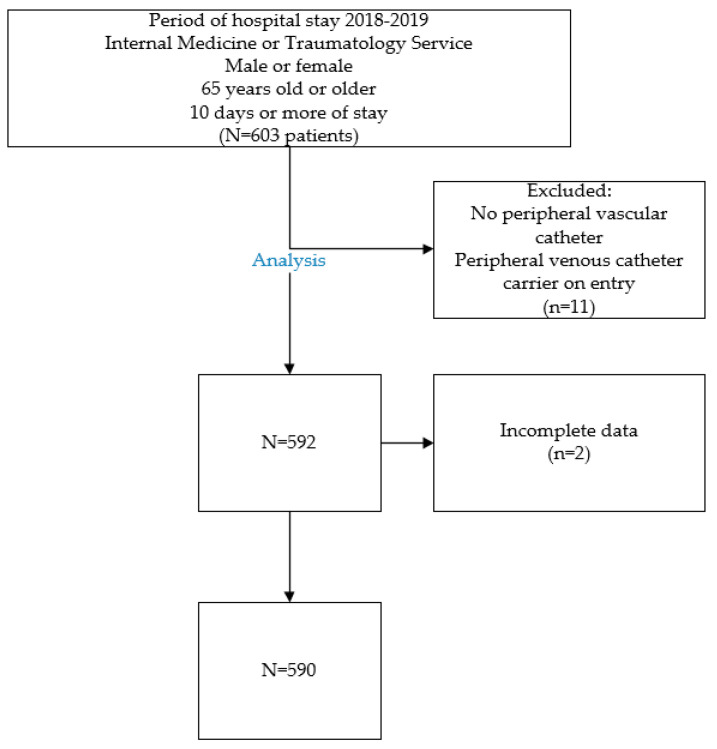
Selection of patients based on inclusion and exclusion criteria.

The flow of inclusion of patients in the study can be seen in Figure 1.

### 2.3. Development of PVC Care and Maintenance Protocol

An expert committee was established, comprising the Nursing Leadership, Nursing Supervisor of Hospitalization Units, and experienced nurses with over 5 years in various departments, including Emergency and Hospitalization. This committee formulated an action protocol based on the latest international standards from the Infusion Nurses Society (INS), good clinical practice guidelines by the Registered Nurses Association of Ontario (RNAO), and recent scientific evidence-based recommendations [6,7,8,9,10].

The nursing protocol established guidelines for the maintenance and care of PVCs. The training of nurses was carried out through an information session explaining the established protocol and the subsequent addressing of any doubts about it. Following a training period for the nursing staff of hospitalization units and a testing phase to address any uncertainties and errors, the protocol’s implementation formally commenced on 1 January 2018. The protocol encompasses the following specific nursing interventions:

1. Hand hygiene practices.
2. Selecting the puncture area and gauge of the PVC (B.Braun Medical, S.A., Madrid, Spain)
3. PVC insertion and securement using a protective or self-adhesive fabric patch.
4. Disinfecting the puncture zone with aqueous chlorhexidine 2% (Bohm laboratories, Madrid, Spain)
5. Documenting PVC gauge, insertion date, and shift.
6. Changing the PVC protective patch at 72 h for acrylate-containing self-adhesive fabric patches, and at 144 h (6 days) for clear adhesive polyurethane patches, including puncture point care.
7. Routine review and replacement of PVC every 144 h (6 days), according to the protocol.
8. Reinitiating the protocol for PVC care and replacement intervals in case of PVC loss, regardless of the cause.

### 2.4. Variables

To assess the impact of adherence to the established protocol on peripheral venous catheter (PVC) loss, the study defined dependent variables and independent variables:

#### 2.4.1. Independent Variables

1. PVC Change Planning: Dichotomous qualitative assessment of whether the catheter change is scheduled at 144 h post-insertion.
2. PVC Change Execution: Dichotomous qualitative measure of whether the PVC is actually replaced at 144 h post-insertion.
3. Dressing Change Planning: Dichotomous qualitative evaluation of whether the dressing change is scheduled as per the protocol.
4. Dressing Change Execution: Dichotomous qualitative assessment of whether the dressing is changed at either 72 or 144 h, depending on the dressing type.

#### 2.4.2. Dependent Variable

Lost PVCs: Dichotomous qualitative measure, excluding cases where removal is due to medical direction or patient action, focusing on PVC loss due to extravasation, phlebitis, or accidental loss.

#### 2.4.3. Control Variable

PVC Lost Before 48 Hours: Dichotomous qualitative assessment of PVC loss within 48 h of insertion. These data may indicate initial problems in the insertion and stabilization of the catheter.

### 2.5. Data Collection Flow

**Figure 2:**
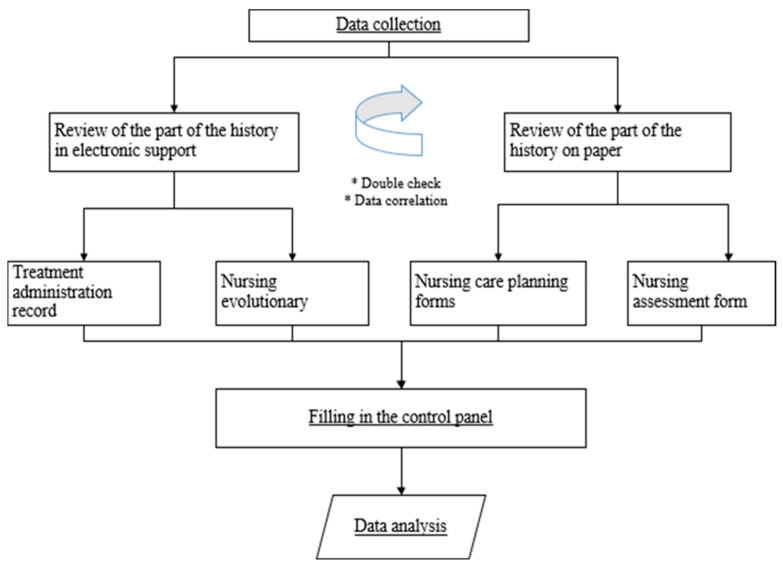
Data analysis. Description of the analysis flow and review of the data from the medical records in paper and computerized format.

The research team from the Quality Department of HM Hospitals Group was responsible for reviewing the medical records of study participants. To enhance the precision of the data collected and address inconsistencies in the completion of various records, the team utilized two distinct sources, ensuring comprehensive and reliable information, as can be seen in Figure 2 *.

This dual verification involved reviewing both nursing evolution and the signature records of treatment administration in the electronic medical records, as well as nursing assessment and care planning.

A dashboard was developed for recording the reviewed data. A skilled nursing team conducted the data analysis. This encompassed information such as PVC placement date, catheter change planning date, puncture site care, dressing change date, and documentation of executed interventions. Also included were details of PVC loss incidents (due to extravasation, infiltration, phlebitis, or accidental loss) if they occurred.

### 2.6. Statistical Analysis

A descriptive statistical analysis was performed using Statistical Package for the Social Sciences International Business Machines IBM SPSS Statistics version 27 software (IBM Corporation USA, Armonk, NY, USA).

Descriptive statistics were performed by means of mean and standard deviation or median and interquartile range, as appropriate. Association between qualitative variables was carried out with the chi-square test or the Fisher exact test, whichever was most suitable for each situation. Statistical significance was established with a *p*-value < 0.05.

### 2.7. Cost Estimation

The Interministerial Commission on Drug Prices establishes the costs of medical supplies [11]. The average cost of PVCs and the costs associated with patients in which the nursing staff complied with the established protocol as well as with those in which it was not complied with were calculated. The average cost of hospital stays in patients based on compliance with the protocol has been calculated. The total average cost refers to the cost of the PVC and stay of each of the patients segmented by the degree of adherence. Total costs of the analyzed procedures were estimated from the payer’s perspective, that is, including the direct healthcare costs associated with the hospital stay and the route change. The cost of the stay was calculated based on published data [12]. The consumed resources were obtained from the analytical accounting of HM Hospitals. This information comes from the Electronic Medical Records of the hospital group (Doctoris^®^), where health resources are charged by minimum consumption units and not by box units. The total cost is obtained by multiplying the number of resource units used by the unit cost. Direct health costs are obtained as the sum of all resources consumed in medical consultations, medical and diagnostic tests, medical material, medications, and resources consumed in surgical procedures. Incremental costs are estimated as the difference between the innovation and the standard considered technique [13]. The costs of materials and hospital stay is described in Table 1.

## 3. Results

### 3.1. Baseline Data for the Study Participants

Baseline data for the study participants are presented in Table 2.

### 3.2. Analysis Based on the Degree of Adherence

**Figure 3:**
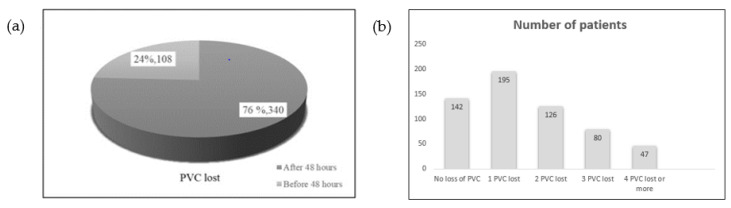
Percentage of PVCs lost before 48 h and distribution of lost PVCs. (**a**) Number of PVCs, with respect to total, lost before and after 48 h from insertion, respectively. (**b**) Distribution by number of patients according to number of lost PVCs.

Data from 590 patients who met all the inclusion criteria and none of the exclusion criteria were analyzed. Among these, 75.93% (448 patients) experienced at least one loss of their PVC during hospitalization and 24.07% (142 patients) did not suffer any PVC loss. Notably, 24% (108 patients) lost their PVC within the first 48 h of placement, as detailed in Figure 3. Furthermore, 56.47% of patients with PVC loss experienced more than one catheter loss during their admission. The total number of PVCs removed due to non-permeability caused by phlebitis, infiltration, extravasation, or loss due to ineffective fixation was 875. The distribution of patients according to the number of lost PVCs is analyzed in Figure 3.

**Figure 4:**
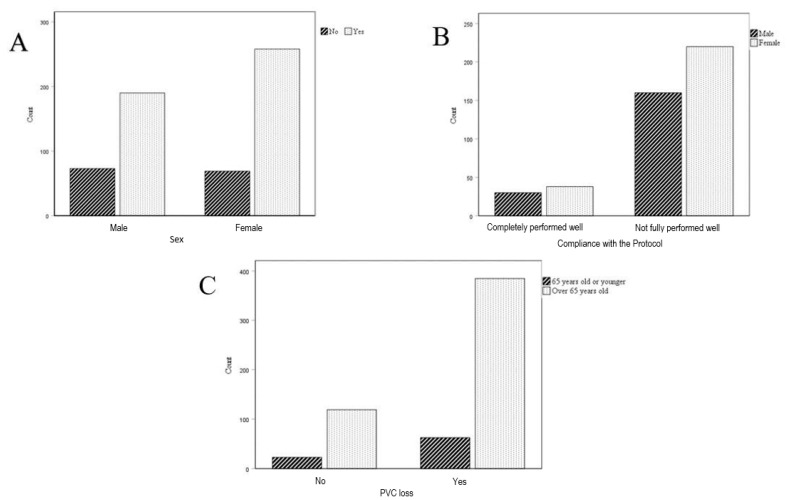
The analysis includes a comparative assessment of PVC loss by gender and across established age groups. It encompasses: (**A**) A comparison of PVC loss between female and male patients; (**B**) an analysis of adherence to PVC care and management protocols based on gender; (**C**) an evaluation of PVC loss in patients over 65 years of age versus those 65 years old or younger.

Among the 448 patients who experienced PVC loss, 190 were male and 258 were female. The loss rate for PVCs was 78.9% in women and 72.2% in men, with no significant gender difference based on the obtained *p*-value (0.060). Additionally, among those with PVC loss where the protocol was not fully followed, 42.1% (160) were male and 57.9% (220) were female. The *p*-value (0.757) indicates no significant gender difference in adherence to the protocol’s stipulations, as depicted in Figure 4.

In the study sample, 85.9% (385 patients) of the patients who experienced PVC loss were over 65 years old, compared to 14.1% (63 patients) for those aged 65 or younger. However, the obtained *p*-value (0.530) suggests no significant difference in PVC loss between patients aged 65 or less and those older than 65, as illustrated in Figure 4.

In the patient sample, 88.8% were under the care of Internal Medicine, with 67% of these patients experiencing PVC loss. Among the 59 patients under Traumatology, 79.6% lost their peripheral venous catheter.

Table 3 presents the percentage of peripheral venous catheter loss in relation to adherence levels to PVC change and care protocols, along with their statistical significance. In patients from Internal Medicine, a significant correlation is observed between adherence to the protocol for line change, dressing change, and maintenance, and a reduced risk of PVC loss. However, in patients from Traumatology, such an association is not evident.

In this study, protocol dictated PVC changes at 144 h post-placement. Out of the 590 patients in the sample, only 19.7% (116 patients) adhered to the guidelines, with 68 patients (15.2%) losing their catheter before 144 h. In 80.3% (474) of the patients in the sample, the nursing professional did not follow the established protocols for the care and maintenance of PVCs, resulting in 84.8% of these patients (380 patients) losing their PVCs, as indicated in Table 4. 

**Figure 5:**
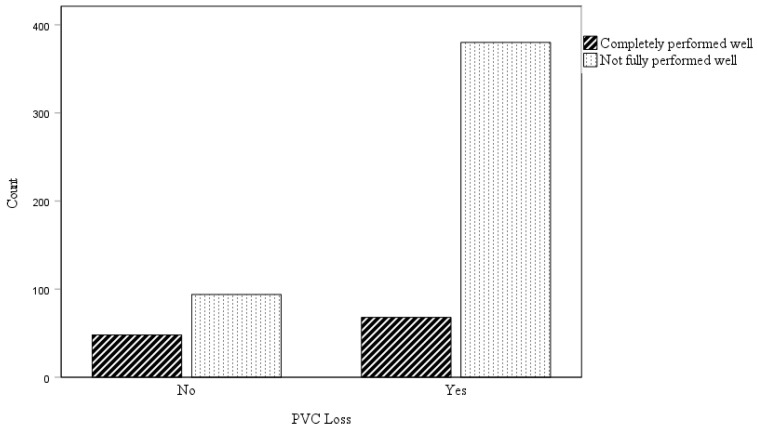
Comparison of PVC loss between patients who complied with the PVC management and care protocol and those who did not.

The *p*-value obtained (<0.05) reveals a statistically significant difference between patients who lost PVCs and adhered to the protocol versus those who lost PVCs and did not comply, as shown in Figure 5.

### 3.3. Cost Comparison Based on Adherence Level

Among the 590 patients in the study, 116 adhered to the protocol, and, of these, 68 patients experienced PVC loss, necessitating 125 new PVC insertions at a total cost of 2771.25 €. In contrast, for the 474 patients who did not follow the protocol, 750 new PVC cannulizations were required, costing 16,627.5 €. The average cost for PVC in patients non-compliant with the protocol was 46.84% higher.

In the study, patients who did not adhere to the PVC protocols accounted for 6423 hospital stays, in contrast to 1293 stays for those who followed the protocols. The average cost of hospital stays was 17.74% higher for patients who did not adhere to the protocols compared to those who did.

As indicated in Table 4, the average total cost for patients who did not adhere to the established protocols was 340.91 €, in comparison to 267.94 € for those who did comply with the protocol.

Table 5 and the data analyzed in this study reveal that the highest incidence of lost peripheral venous catheters (PVCs) occurs in hospital admissions ranging from 10 to 30 days, which also correspond to the periods with the highest number of hospital stays.

## 4. Discussion

The main results obtained from this study are the following:

1. In 80.3% (474) of the patients in the sample, the nursing professional did not follow the established protocols for the care and maintenance of PVCs, resulting in 84.8% of these patients (380 patients) losing their PVCs.
2. A total of 75.93% of the patients who participated in the study lost at least one PVC, and 24% of these lost it before 48 h.

This study evaluated the effect of protocol adherence on catheter loss, including accidental loss, extravasation, infiltration, and phlebitis, in PVC care. Over 70% of patients, and up to 81% of those in internal medicine, use PVCs [2,14].

The US Centers for Disease Control and Prevention (CDC) recommend implementing effective strategies and thoroughly advocate for effective strategies and thorough documentation to ensure safe care and reduce adverse events. However, data on the impact of adherence to these standards is limited, as highlighted by the Venous Thromboembolism Zero Program. The ENEAS (2005) study further indicates that recommended practices are not fully implemented in hospitals, with the avoidability of adverse effects ranging from 50% to 70% [4,5,15].

The study underscores the significant impact of training, experience, and evidence-based practice by healthcare professionals on the incidence of adverse effects and complications related to PVC cannulation. It highlights the unacceptably high rate of PVC loss, impacting patient safety. The observed variability in care delivery underscores the lack of effective measures and evidence of compliance with and adherence to established recommendations and standards [5,6].

PVC loss occurred in 75% of cases, with phlebitis identified as one of the potential causes. This rate aligns with findings from other published studies but is higher than some reports which indicate PVC loss rates between 57.8% and 54.05% [15,16,17].

Phlebitis is recognized as the primary cause of catheter failure, with incidence rates ranging between 30.7% and 44% in various studies [18,19,20]. Despite some studies reporting lower phlebitis rates, all significantly exceed the 5% prevalence rate recommended by the Infusion Nurses Society (INS) [3,21]. The findings of this study indicate that 14% of PVC loss occurred within the first 24 h and 25% within 48 h. These figures are lower compared to other studies, which report PVC loss exceeding 55% within these time frames. The average duration of PVC usage noted in this research ranges between 2.5 to 4 days (60 to 96 h) [14,15,16,19,20,22].

While most studies indicate an average hospital stay of no more than 3.5 days, the specific risks and common complications beyond this period remain unclear [23]. Current standards and recommendations, including those for detecting microorganisms in case of infection, aim to ensure safe practice and promote a culture of safety, emphasizing daily monitoring to reduce adverse effects [6,7,24,25]. Studies have shown a significant difference in catheter failure rates within the first 38 h, decreasing over time, highlighting the importance of daily follow-up [6,7,16,24]. Adequate nurse training in the latest guidelines and scientific evidence is crucial, as is the implementation of standards [26]. Monitoring staff interventions is essential to ensuring adherence to standards and early complication detection [27]. Compliance with standards significantly impacts the PVC loss rate, potentially reducing it by up to 11% [28].

The study emphasizes that implementing best practice guidelines is crucial for risk management and reducing phlebitis rates [7,29,30,31]. The effectiveness of these guidelines’ implementation is a key factor. PVC loss significantly affects patient safety, potentially causing treatment delays, repeated cannulation pain, and increased costs and nursing time. In this study, 10.5% of patients required four or more new cannulations due to PVC loss, compared to other studies reporting up to 18.2% of patients needing five to nine cannulations for the same reason [31].

While some studies suggest an increased rate of catheter loss with clinically indicated removal versus routine replacement, numerous publications indicate no significant difference in PVC complications between the two methods [8,20,21,22,32,33,34]. More research is needed to understand the impact of these practices on bloodstream infection rates. Implementing and adhering to good practice guidelines for PVC management is essential to reducing bloodstream infections associated with PVC use [35,36,37,38,39,40,41,42]. Establishing concrete quality indicators for monitoring, along with maintaining training and evidence-based care, is crucial [40,42,43]. This study demonstrates the need for accurately recording nursing interventions, which in some cases were less than 50%. Such meticulous record keeping is vital for adequate follow-up in the care and maintenance of PVCs [34,44,45,46].

Designing effective training and reviewing adherence to standards based on current evidence is crucial, as is understanding nurse adherence rates to established protocols [34]. Deviations in nursing interventions, such as the use of transparent dressings or the daily review of puncture sites, have been noted [47]. This study’s findings align with these observations, revealing that in 68% of cases where PVCs were lost, the recommendations for follow-up and dressing change were not followed.

Formative intervention and training of nurses, coupled with ensuring their awareness of the latest scientific evidence, leads to better adherence to the most current guidelines. This approach results in a notable improvement in practices based on established evidence and adherence to set standards [48,49]. Adherence to protocols for PVC insertion varies across different hospital services. Despite similar rates of complications, varying factors in different services affect adherence, often leading to a failure in meeting patient safety principles and standards established by recent scientific evidence [6,7,14,45]. This study concludes that adherence to evidence-based standards significantly impacts PVC loss. While the results exceed INS guidelines, the emphasis is not solely on instructing best practices but also on implementing and monitoring these practices to identify and rectify deviations in deployment and compliance [6]. Based on this study’s findings, it is crucial to pay special attention to and further investigate the evolution and complications of PVCs between 96 and 144 h. Considering the average PVC duration ranges from 2.5 to 4 days, this research suggests the need for specific measures to reduce PVC loss and extend its average lifespan.

The lack of identification of the specific causes of PVC loss makes it difficult to implement effective preventive measures, and could be a line of research for future studies. In addition, possible staff turnover must be taken into account as well as the training and education of nurses adapted to PVC maintenance and care protocols.

## 5. Conclusions

This study highlights the significant impact of protocol adherence on peripheral venous catheter loss and patient safety. It reveals that non-adherence to PVC care protocols leads to a higher rate of catheter loss and increased healthcare costs. The findings underscore the need for rigorous implementation and monitoring of established catheter care protocols, emphasizing the role of nursing in improving patient outcomes and reducing healthcare expenses. These conclusions point towards the importance of ongoing education and protocol compliance in nursing practice to enhance patient safety. It is crucial to establish an integrated, continuous, in-service training program that withstands personnel fluctuations and ensures constant updating of knowledge and skills, as well as adherence to good practice protocols and guides.

## Figures and Tables

**Table 1:** Costs of materials used for PVC piping and costs of hospital stay.

	PVP
List of material needed for PVC	
Needle to load	0.26 €
Tegaderm dressing 7 × 8 cm	2.99 €
Aqueous chlorhexidine 2%	2.34 €
Transpore sponges 2.5 × 10	1.73 €
Sterile gauze 1 pack	0.47 €
Peripheral venous catheter	4.60 €
Syringes 5 cc	0.32 €
Mefix sponges 10	1.65 €
3-step wrench with/without extension	3.68 €
Paper sponges 2.5 × 10	0.88 €
Physiological saline solution 10 cc	0.21 €
Heparinized stoppers	2.34 €
Fabric sponges 5 × 2.5	0.70 €
Total PVC cost	22.17 €
Hospital stay	409.61 €

**Table 2:** Baseline characteristics of the patients included and sample percentage.

N			590
		Absolute values	%
Sex (%)	Male	263	45
	Female	327	55
Average age (%)	65 years or younger	86	15
	Over 65 years old	504	85
Average length of stay (%)	Between 10 and 30 days	556	94
	Between 31 and 50 days	28	5
	More than 50 days	6	1
Year of entry (5)	2018	305	52
	2019	285	48
Hospital discharge Service (%)	Internal Medicine	524	89
	Traumatology	66	11

**Table 3:** Percentage of PVC (peripheral venous catheter) loss depending on the degree of adherence to the PVC change protocol and *p*-value obtained. Percentage of PVC loss depending on the degree of adherence to the PVC dressing change and care protocol. Comparison of results as a function of adherence to the indications given.

	Protocol Regarding the Change of PVC					Protocol for the Care and Change of PVC Dressing				
	Failure to Comply with Protocol		Protocol Compliance		*p* Value	Failure to Comply with Protocol		Protocol Compliance		*p* Value
	Loss of PVC	No Loss of PVC	Loss of PVC	No Loss of PVC		Loss of PVC	No Loss of PVC	Loss of PVC	No Loss of PVC	
**Total of** **the sample**	79.3% (291)	20.7 % (76)	62.9% (110)	37.1% (291)	<0.001	78.4% (291)	21.6% (80)	68.8% (137)	31.2% (62)	0.012
**Men**	74.4% (116)	25.6% (40)	62.8% (54)	37.2% (32)	0.060	77.2% (129)	22.8 (38)	59.8% (52)	40.2% (35)	0.003
**Women**	82.9% (175)	17.1% (36)	62.9% 56)	37.1% (33)	<0.001	79.4% (162)	20.6% (42)	75.9% (85)	24.1% (27)	0.469
**% 65 years old or Younger**	75.9% (44)	24.1% (4)	52.6% (10)	47.4% (9)	0.055	79.6% (43)	20.4% (11)	55.6% (15)	44.4% (12)	0.024
**% over 65 years old**	79.9% (247)	20.1% (62)	64.1% (100)	35.9% (56)	<0.001	78.2% (248)	21.8% (69)	70.9% (122)	29.1% (50)	0.072
**Internal** **Medicine** **Service**	78.7% (255)	21.3% (69)	62.3% (99)	37.7% (60)	<0.001	77.7% (255)	22.3% (73)	68.5% (124)	31.5% (57)	0.020
**Traumatology** **Service**	83.7% (36)	16.3% (7)	68.8% (11)	31.3% (5)	0.204	83.7% (36)	16.3% (7)	72.2% (13)	27.8% (5)	0.303

**Table 4:** Comparison of costs based on compliance or non-compliance with the PVC management protocols.

	Compliance with the Protocol Established for PVC	Non-Compliance with the Protocol Established for PVC	
**Total sample**	116	474	
Patients who have lost PVCs	68	380	
N° of PVCs channeled (united)	125	750	
Total cost of PVCs	2771.25 €	16,627.50 €	
Average cost of PVCs	23.89 €	35.08 €	46.84% *
Cost of not complying with the protocol (22.17 euros per lane)		13,856.25 €	
Total number of stays	1987	7766	
Total stays of patients who lost their PVCs	1293	6423	
Total stays of patients who lost their PVCs	4565.74 €	5550.47 €	17.74% **
Total average cost	267.94 €	340.92 €	21.41% ***

* The mean cost of PVCs was 46.84% higher in patients who did not comply with the protocols compared to those who did. ** The average cost of stays was 17.74% higher in patients who did not comply with the protocols compared to those who did. *** The average total cost was 21.41% higher in patients who did not comply with the protocols compared to those who did.

**Table 5:** Relationship between hospital stay and lost PVCs depending on adherence or non-adherence to the established protocol.

	Number of Patients in Relation to the Number of Lost PVCs								Number of Patients in Relation to the Number of Lost PVCs							
Hospital Stay	Adherence to Protocol (*)	Patients Who Lost PVC	1 PVC	2 PVC	3 PVC	4 PVC	Total PVCs Lost and Requiring Re-Routing	Total Number of Stays	Non-Adherence to Protocol (**)	Patients Who Lost PVC	1 PVC	2 PVC	3 PVC	4 PVC	Total PVCs Lost and Requiring Re-Routing	Total Number of Stays
From 10 a 20 days	89	47	29	10	4	4	77	630	374	290	140	93	40	17	514	3869
From 21 a 30 days	22	16	6	3	5	2	35	427	92	82	17	19	27	19	212	2115
From 31 a 40 days	0	0	0	0	0	0	0	0	0	0	0	0	0	0	0	0
From 41 a 50 days	5	5	2	0	1	2	13	236	2	2	1	0	1	0	4	93
From 51 a 60 days	0	0					0	0	4	4	0	1	2	1	12	217
From 61 a 70 days	0	0						0	2	2	0	0	0	2	8	129
Total	116	68					125	1293	474	380					750	6423
**Total PVC piped**		**875**														

* Of the patients who complied with the established protocol, 89 had a stay of 10 to 20 days and 77 PVCs had to be channeled; 22 patients had a stay of 21 to 30 days and 35 PVCs had to be channeled. ** Of the patients who did not comply with the established protocol, 290 had a stay of 10 to 20 days and 514 PVCs had to be channeled; 82 patients had a stay of 21 to 30 days and 212 PVCs had to be channeled.

## Data Availability

The data supporting the results and findings of this study are available from the corresponding author. These data, due to confidentiality and ethical considerations, are not publicly available.
